# Enhancing Mechanisms of the Plant Growth-Promoting Bacterial Strain *Brevibacillus* sp. SR-9 on Cadmium Enrichment in Sweet Sorghum by Metagenomic and Transcriptomic Analysis

**DOI:** 10.3390/ijerph192316309

**Published:** 2022-12-06

**Authors:** Xiao-Qi Li, Yong-Qi Liu, Ying-Jun Li, Hui Han, Hao Zhang, Ming-Fei Ji, Zhao-Jin Chen

**Affiliations:** Collaborative Innovation Center of Water Security for Water Source Region of Middle Route Project of South-North Water Diversion in Henan Province, College of Water Resource and Environment Engineering, Nanyang Normal University, Nanyang 473061, China

**Keywords:** sweet sorghum, plant growth-promoting bacteria, Cd, metagenome, transcriptome

## Abstract

To explore the mechanism by which the plant growth-promoting bacterium *Brevibacillus* sp. SR-9 improves sweet sorghum tolerance and enriches soil cadmium (Cd) under pot conditions, the effect of strain SR-9 inoculation on the microbial community of sorghum rhizosphere soil was analyzed by metagenomics. Gene expression in sweet sorghum roots was analyzed using transcriptomics. The results showed that strain SR-9 promoted the growth of sweet sorghum and improved the absorption and enrichment of Cd in the plants. Compared with the uninoculated treatment, the aboveground part and root dry weight in strain SR-9 inoculated with sorghum increased by 21.09% and 17.37%, respectively, and the accumulation of Cd increased by 135% and 53.41%, respectively. High-throughput sequencing showed that strain SR-9 inoculation altered the rhizosphere bacterial community, significantly increasing the relative abundance of Actinobacteria and Firmicutes. Metagenomic analysis showed that after inoculation with strain SR-9, the abundance of genes involved in amino acid transport metabolism, energy generation and conversion, and carbohydrate transport metabolism increased. KEGG functional classification showed that inoculation with strain SR-9 increased the abundance of genes involved in soil microbial metabolic pathways in the rhizosphere soil of sweet sorghum and the activity of soil bacteria. Transcriptome analysis identified 198 upregulated differentially expressed genes in sweet sorghum inoculated with strain SR-9, including those involved in genetic information processing, biological system, metabolism, environmental information processing, cellular process, and human disease. Most of the annotated differentially expressed genes were enriched in the metabolic category and were related to pathways such as signal transduction, carbohydrate metabolism, amino acid metabolism, and biosynthesis of other secondary metabolites. This study showed that plant growth-promoting bacteria can alter the rhizosphere bacterial community composition, increasing the activity of soil bacteria and upregulating gene expression in sweet sorghum roots. The findings enhance our understanding of the microbiological and botanical mechanisms by which plant growth-promoting bacterial inoculation improves the remediation of heavy metals by sorghum.

## 1. Introduction

With the rapid development of agricultural inputs, urbanization, and the economy, soil heavy metal pollution is becoming increasingly serious [1,2,3]. Yuan et al. [4] evaluated the level of heavy metal contamination in Chinese soils using meta-analysis based on papers on heavy metal contamination in agricultural and urban soils published in China from 2000 to 2019. The results showed that Cd was the most abundant heavy metal polluting Chinese soils, and the average Cd concentration in Chinese soils was 0.19 mg kg^−1^, with the Cd concentrations in agricultural and urban soils being 0.19 and 0.29 mg kg^−1^, respectively; these values were two and three times higher than the background level of Cd (0.097 mg kg^−1^), respectively. Cd in soil shows high environmental migration and is easily absorbed and accumulated by plants, thus affecting plant growth and development by weakening photosynthesis, interfering with the absorption and assimilation of nutrient elements, destroying plant metabolism, and inducing leaf yellowing [5,6,7]. In addition, Cd reduces plant biomass and accumulates in edible parts, eventually causing serious harm to human health through the food chain [8]. Cd entering the human body may cause physiological damage to various organs, such as the lung, kidney, placenta, and bone, and lead to many diseases [9]. Therefore, control and remediation of soil Cd pollution is an urgent issue. Phytoremediation, given its advantages of environmental friendliness, low cost, suitability for large-scale use, and sustainability is widely used in the remediation of heavy metal-contaminated soil [10,11]. Phytoremediation technology mainly includes the use of plants and related soil microorganisms to extract heavy metals from soil and the use of plant roots to enhance the fixation of heavy metals [12,13].

Plant growth-promoting bacteria (PGPB) are bacteria that do not cause damage to plants but rather have a positive impact on their growth and development. PGPB are usually found in the rhizosphere soil or in plants and are often used in phytoremediation to improve the efficiency of remediation [14,15]. Numerous studies have shown that PGPB in soil promote plant growth through various mechanisms, including the production of phytohormones (indoleacetic acid, IAA) and siderophores, alteration of 1-aminocyclopropane-1-carboxylic acid deaminase activity (ACC), and dissolved phosphorus and nitrogen fixation, as well as changes in the rhizosphere microbial environment, to improve the development and growth of plants under adverse conditions and their ability to accumulate heavy metals and enhance the efficiency of the phytoremediation of soil heavy metals [16]. Therefore, plant-microbial combined remediation has become a trending topic in remediation research. In recent years, due to the unique advantages of energy plants, they have received increasing attention in the remediation of heavy metal-contaminated soils. The selection of energy plants as remediation plants can not only combine soil remediation with agricultural energy production and relieve the competition for farmland between energy crops and food crops but also transfer heavy metals from the food chain to the energy chain, which has broad application prospects [17,18]. Sweet sorghum belongs to the C4 grass family, has a high photosynthetic efficiency and high biomass, is the only crop that yields both grains and stems, can be used as a raw material for the production of ethanol, and has been identified by the U.S. Department of Energy as one of the important herbaceous energy crops [19,20]. In recent years, studies have confirmed that sorghum or sweet sorghum has the ability to absorb and accumulate heavy metals and is more resistant to Cd treatment than wheat and maize [21,22]. At the same time, studies have shown that PGPB inoculation can improve sorghum growth and heavy metal accumulation, and the biomass of sweet sorghum inoculated with the plant growth-promoting endophyte SLS18 in Cd-contaminated soil increased by 38% compared with that of the control group [17]. The inoculation of three heavy metal-tolerant PGPB by EI-Meihy et al [23] had a good improvement and promotion effect on the growth of sorghum seedlings treated with Cd, Cu, Pb, and Zn stress and alleviated the toxic effects of the heavy metals.

In recent years, with the development of omics technology (metagenomics, transcriptomics, etc.), such technology has been applied in plant-microbial combined remediation because of its technical advantages and has helped to identify PGPB as being able to improve the microbial and botanical mechanisms of plant repair. Few studies have revealed soil function in terms of microbial community structure, diversity, and function under joint plant-microbial remediation, and the gene function changes that occur in sweet sorghum grown under heavy metal pollution are not clear. Therefore, this study used 16S rRNA gene metabarcode sequencing, metagenome sequencing technology, and transcriptomics technology to study the microbiological and botanical mechanisms by which inoculation of the plant-growth-promoting bacterial strain *Brevibacillus* sp. SR-9 improves the growth and accumulation of Cd in sweet sorghum.

## 2. Materials and Methods

### 2.1. PGPB and Cd-Contaminated Soil

The soil used in this experiment was topsoil (0–20 cm) collected in West District Agricultural Park of Nanyang Normal University. The soil was air-dried and passed through a 4 mm sieve, and analytically pure 3CdSO_4_·8H_2_O was added to bring the soil Cd^2+^ content to 20 mg·kg^−1^. The mixture was stirred well and left to sit for a month to allow the soil to fully absorb and fix Cd^2+^. The strain *Brevibacillus* sp. SR-9 (SUB11374180) was isolated from Cd-contaminated farmland soil in Nanyang, China, which was stored in our laboratory, and shows high tolerance to heavy metals, siderophore production, IAA production, phosphorus and potassium dissolution, and other growth-promoting properties. The fermentation broth of the strain was centrifuged at 3900 r·min^−1^ for 20 min, the supernatant was decanted, and the bacterial precipitate was collected, washed with 0.9% NaCl solution 3 times, and resuspended in sterile deionized water to achieve a thallus concentration of OD600 = 1.0; this was the bacterial suspension used for inoculation.

### 2.2. Potted Plant Experimental Design and Treatments

Two treatment groups were set up in this experiment: G20 (uninoculated; Cd^2+^ concentration, 20 mg·kg^−1^) and G20S (inoculated with strain SR-9; Cd^2+^ concentration, 20 mg·kg^−1^). There were 12 replicates per treatment for a total of 24 samples. The sweet sorghum seeds selected were from the Henan conventional sweet sorghum cultivar Hercules and were disinfected with 75% alcohol for 5 min and washed several times with deionized water. A total of 2.5 kg of soil was placed in each pot (20 cm diameter × 15 cm height), 8 seeds were evenly sown per pot, and a thin layer of soil was spread over the seeds. After the sorghum seeds emerged, the seedlings were gradually thinned out by selecting only seedlings with similar and vigorous growth. Three sorghum seedlings were left in each pot, and all pots were arranged randomly. At days 30, 50, and 70, 30 mL of the bacterial suspension was poured into the roots of the sweet sorghum seedlings, and an equal volume of sterile deionized water was added to a blank control without bacteria. The pots containing plants were completely randomized and maintained in a greenhouse. Management of sorghum followed traditional methods, such as maintaining the soil moisture content and controlling humidity and temperature as the sorghum grew.

Three months after planting, the sorghum plants were harvested. The sorghum plants were divided into two parts, roots, and stems and leaves, and fixed at 105 °C for 20 min. Next, they were dried at 70 °C to constant weight, and the dry weight was determined. The dried samples were pulverized by a grinder and digested by the HNO_3_-HClO_4_ method (1:3 volume ratio), followed by measurement of Cd content by inductively coupled plasma–optical emission spectroscopy (ICP-OES). The limit of detection (LOD) and the limit of quantification (LOQ) were 0.003 and 0.009 μg mL^−1^, respectively. Plant (GBW-10012) and soil (GBW-08303) standard reference materials were provided by the National Research Center for Certified Reference Materials (Beijing, China) and examined to validate the method used. The Cd recovery rates were 95 ± 6% for GBW10012 and 94 ± 7% for GBW-08303.

### 2.3. Collection of Soil Samples

The loosely bound rhizosphere soil of each treatment (12 biological replicates for each sample) was obtained by gently shaking the roots, while the rhizosphere soil adhering to the root system was isolated by more vigorous shaking or by hand. Some of the rhizosphere soil was placed in sealed bags and stored at −80 °C. The remaining rhizosphere soil was allowed to dry at room temperature, and additional indices were measured [24].

### 2.4. Analysis of the Soil Microbiota Composition

A FastDNA^®^ Spin Kit for Soil (MP Biomedicals, Irvine, CA, USA) was used to extract the total DNA of sorghum rhizosphere soil in each treatment, and PCR amplification was performed using the universal bacterial primers 338F/806R. The amplification conditions followed the protocol of Chen et al. [25]. The DNA concentration and purity were determined, and the samples were then sent to Maggi Biomedical Technology Co., Ltd. (Shanghai, China) for sequence determination using a MiSeq PE300 sequencer (Illumina Inc., San Diego, CA, USA). We performed quality control of the original sequence and obtained valid data. Taxonomy was assigned using the SILVA database (v. 138) with QIIME2 software (v. 2020.11). Data analysis was performed online using the Meiji Biological Cloud Platform (https://cloud.majorbio.com/).

### 2.5. Metagenomic Analysis

Soil samples weighing 0.5 g were obtained, and the total DNA was extracted from the sweet sorghum rhizosphere soil samples according to the instructions of the PowerWater DNA Extraction Kit. The concentration of DNA was detected by TBS-380, the purity of metagenomic DNA was quantitatively detected by a Nano Drop200 UV spectrophotometer, and the integrity of the DNA was measured by 1% agarose gel electrophoresis. Two-microgram samples of high-quality DNA were stored in the refrigerator at −80 °C for testing [26].

The HiSeq 2000 sequencing platform provided by Shanghai Meiji Biomedical Cloud Platform was used for the metagenomic sequencing of sweet sorghum rhizosphere soil samples. The original sequence obtained on the sequencing platform was quality-controlled with Fastp v0.20.0 software, low-quality reads were removed from the data to obtain high-quality sequences, and MEGAHIT v1.1.2 software was used for sequence assembly. ORF gene prediction was performed on the splicing results, and the predicted results were clustered to establish the nonredundant gene set. The nonredundant gene set sequences were aligned with the eggNOG database using DIAMOND (https://github.com/bbuchfink/diamond) (parameters: blastp; E-value ≤ 1 × 10^−5^) to obtain the clusters of orthologous groups (COGs), and their abundances were calculated using the sum of the gene abundances corresponding to the COGs.

### 2.6. Transcriptome Analysis of Sorghum Roots

Total RNA from sorghum roots was extracted with TRIzol reagent (Invitrogen, Carlsbad, CA, USA), and high-quality RNA samples were selected for high-throughput sequencing. The Meiji Biological Cloud Platform was commissioned to complete the transcriptome sequencing. High-quality clean reads were obtained after quality control, sequenced, and compared with the reference genome of *Sorghum bicolor* using HISAT2 software to obtain mapped reads. The mapped reads were spliced using String Tie based on the selected reference genome sequence, and the genes were compared with six databases, GO, Swiss-Prot, COG, NR, KEGG, and KOG. The annotation information of genes was obtained, the annotation information from each database was statistically analyzed, and the gene expression level was quantitatively analyzed.

### 2.7. Data Analysis

The data from the treatments were compared by analysis of variance and Tukey’s test at a 5% significance level (*p* < 0.05) in SPSS V. 19.0 for Windows. Mathematically processed results are presented in the form M ± SE, where M is the arithmetic mean and SE is the standard error.

## 3. Results and Analysis

### 3.1. Effects of Brevibacillus SR-9 Inoculation on Sorghum Biomass and Cd Accumulation

**Figure 1:**
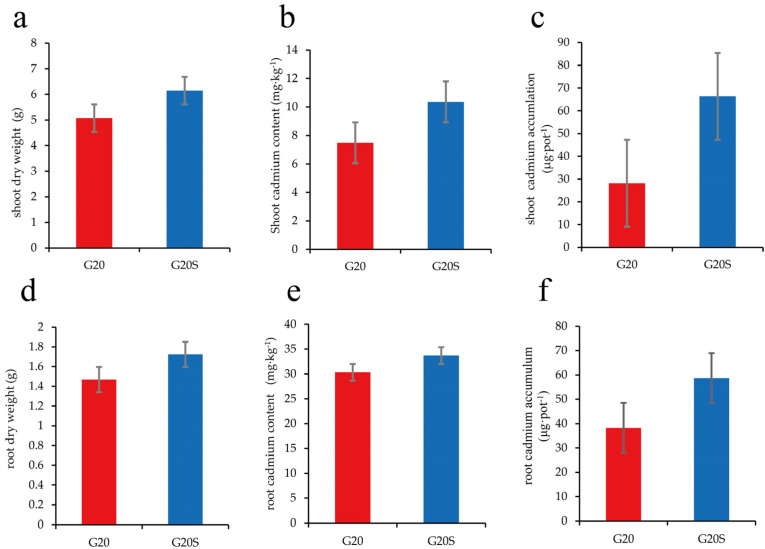
Sweet sorghum biomass and Cd accumulation under the G20 (noninoculation) and G20S (strain SR-9 inoculation) treatments: (**a**) shoot dry weight (**b**) shoot Cd content (**c**) shoot Cd accumulation (**d**) root dry weight (**e**) root Cd content, and (**f**) root Cd accumulation.

The dry weight and heavy metal content of sorghum shoots and roots are shown in Figure 1. Compared with the noninoculation treatment, strain SR-9 promoted the growth of sweet sorghum plants, and the dry weight of the aboveground parts and roots increased by 21.09% and 17.37%, respectively. The results showed that compared with those under the noninoculation treatment, the Cd content in the aboveground parts and roots under the strain SR-9 inoculation treatment increased by 38.53% and 11.13%, respectively. The Cd content in the roots of sorghum was much higher than in the shoots. The Cd concentration in the noninoculation and strain SR-9 inoculation treatments was 28.17 μg·pot^−1^ and 66.34 μg·pot^−1^ in the shoots and 38.25 μg·pot^−1^ and 58.68 μg·pot^−1^ in the belowground parts, respectively. Compared with the noninoculation treatment, the accumulation of Cd in the aboveground parts and roots of inoculated sorghum increased by 135% and 53.41%, respectively. In addition, the Cd transfer coefficient and Cd enrichment coefficient of sorghum inoculated with strain SR-9 increased by 24%, 40.54% (shoot), and 10.53% (root), respectively, compared with those of sorghum without strain SR-9 inoculation, which indicated that strain SR-9 inoculation could improve the ability of sweet sorghum to remediate soil Cd.

### 3.2. Microbial Community Composition

**Figure 2:**
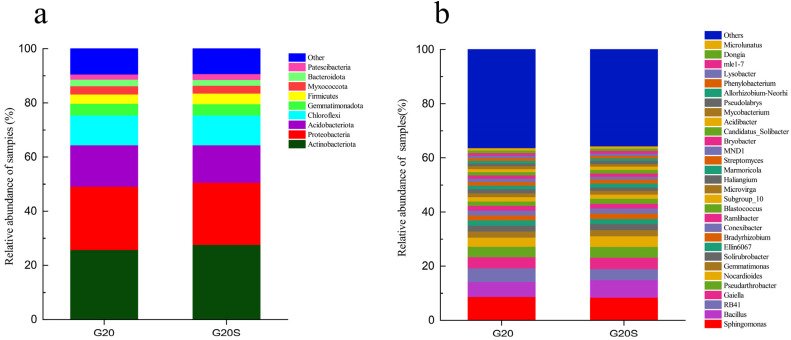
Bacterial community structure of sweet sorghum rhizosphere soil under the G20 (noninoculation) and G20S (strain SR-9 inoculation) treatments: (**a**) phylum-level analysis (**b**) genus-level analysis.

The results of high-throughput sequencing showed that the bacteria in the rhizosphere soil samples of sweet sorghum were composed of 38 phyla and 934 genera. Phylum-level analysis showed that the rhizosphere soil bacterial communities of sweet sorghum in the two treatment groups mainly (relative abundance > 1%) consisted of nine gates. Actinobacteriota (26.95–28.91%), Proteobacteria (24.06–24.46%), Acidobacteriota (14.52–16.02%), Chloroflexi (11.51–11.54%), Gemmatimonadota (4.31–4.47%), and Firmicutes (3.59–4.08%) were the dominant phyla (Figure 2a). Compared with the noninoculation treatment, strain SR-9 inoculation significantly increased the relative abundance of Actinobacteria (1.99%) and Firmicutes (0.49%), and significantly decreased the relative abundance of Proteobacteria (0.4%) and Acidobacteria (1.5%). The 20 most abundant populations at the genus level were analyzed. *Sphingomonas*, *Bacillus*, *Pseudarthrobacter*, *Bradyrhizobium* and *Streptomyces* were some of the most abundant genera in the samples (Figure 2b).

**Figure 3:**
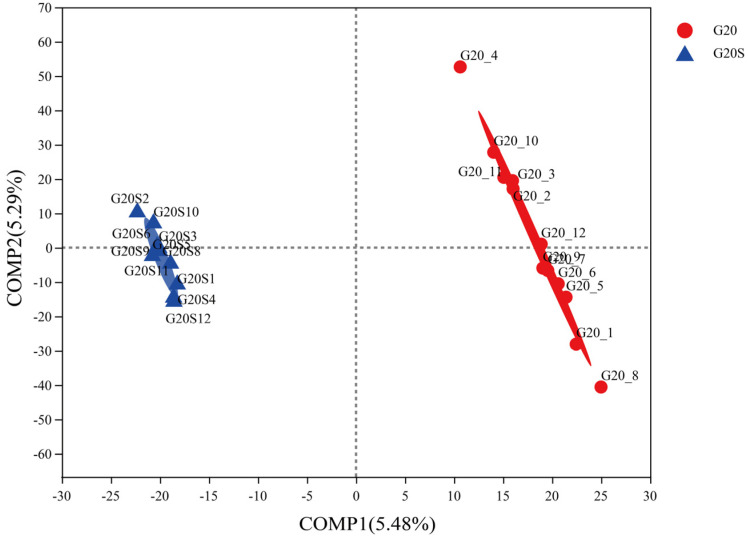
Differences in bacterial community structure between the G20 (noninoculation) and G20S (strain SR-9 inoculation) treatments based on PLS-DA.

**Figure 4:**
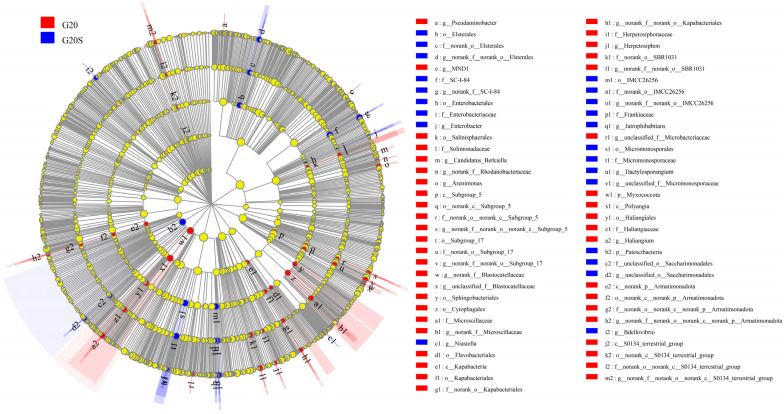
Differential bacterial distribution of the G20 (noninoculation) and G20S (strain SR-9 inoculation) treatments.

The differences in bacterial community structure between the two groups were examined by partial least squares-discriminant analysis (PLS-DA). Figure 3 shows that at the OTU level, the uninoculated and strain SR-9 inoculated samples are clustered at either end of the graph, indicating differences between the uninoculated and strain SR-9 inoculated groups. In terms of sample segregation, the bacterial community composition under strain SR-9 inoculation did not change much, whereas, in the uninoculated group, the bacterial community composition was more distinct. LEfSe software was used to find biomarkers with significant differences between sorghum rhizosphere bacterial communities. A total of 35 bacterial genera differed with a linear discriminant analysis (LDA) score of ≥2.0, mainly in the Micrococcus phylum (24 genera) (Figure 4). The abundance of significantly different bacteria in the G20S treatment group inoculated with strain SR-9 was significantly lower than that in the uninoculated treatment group.

### 3.3. Metagenomic Analysis

**Figure 5:**
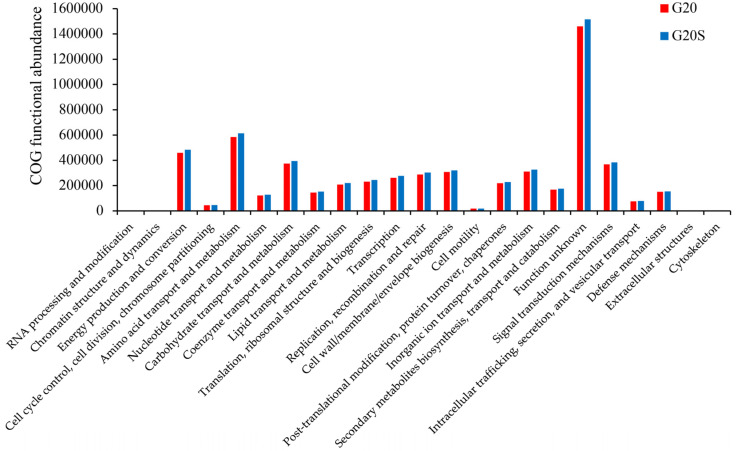
Functional annotation of microbial COG in rhizosphere soil of sweet sorghum between the G20 (noninoculation) and G20S (strain SR-9 inoculation) treatments.

Metagenomic sequencing technology was used to analyze the microbial functions of rhizosphere soil samples from the two treatment groups, and four major categories were annotated in COG, which were refined into 23 functional groups (Figure 5). Cellular Processes and Signaling contained nine functional categories, with the highest abundance found for signal transduction mechanisms and the lowest abundance for extracellular structures. In information storage and processing, the abundance of replication, recombination, and repair was the highest, while the abundance of RNA processing modification was the lowest. In addition to the unknown functional classification, the total abundance of Metabolism was highest in the two groups of samples. Genes involved in amino acid transport metabolism, energy generation and conversion, and carbohydrate transport metabolism were the top three functional genes with the highest relative abundance. Therefore, amino acid metabolism and energy generation and conversion were the most important processes in sweet sorghum rhizosphere soil microorganisms.

**Figure 6:**
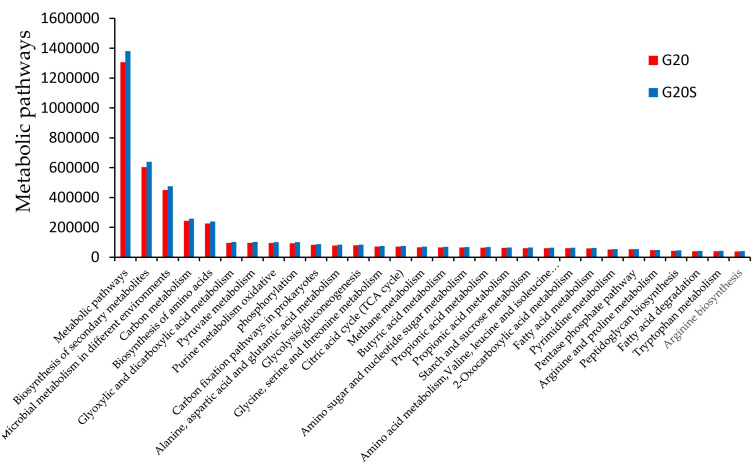
Abundance map of the top 30 tertiary metabolic pathways annotated by KEGG between the G20 (noninoculation) and G20S (strain SR-9 inoculation) treatments.

Combined with the GO database, KEGG functional classification was conducted for the genes of sweet sorghum rhizosphere soil microorganisms under two different treatments. The KEGG secondary classification results showed that the metabolic genes in the two treatment groups were the highest, which was consistent with the COG annotation results. The top 30 tertiary metabolic pathways were selected for analysis (Figure 6). It was noted that the metabolic pathway (KO01100) had the highest abundance, followed by the biosynthesis of secondary metabolites (KO01110), and the abundance of all metabolites in the strain SR-9 inoculation treatment was higher than that in the noninoculation treatment. The results showed that after inoculation with strain SR-9, the abundance of soil microbial metabolic pathways in the rhizosphere soil of sweet sorghum increased, and the activity of soil bacteria also increased.

**Figure 7:**
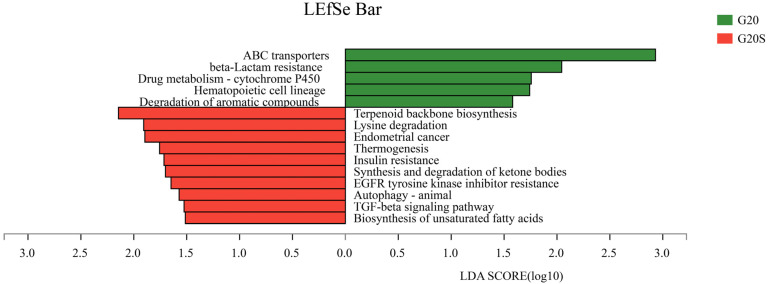
Differential analysis of KEGG metabolic pathways between the G20 (noninoculation) and G20S (strain SR-9 inoculation) treatments.

To screen metabolic pathways with significant differences between groups and detect differential functions among different groups, LDA was used to achieve dimensionality reduction and evaluate the impact of differential functions; that is, the LDA score was obtained. As shown in Figure 7, among the tertiary metabolic pathways, significantly different pathways in the strain SR-9 inoculated sample included terpenoid backbone biosynthesis, lysine degradation, biosynthesis of unsaturated fatty acids, TGF-beta signaling pathway, and thermogenesis. The significant pathways in the uninoculated sample were ABC transporters, beta-lactam resistance, drug metabolism-cytochrome P450, hematopoietic cell lineage, and degradation of aromatic compounds.

### 3.4. Transcriptome Gene Expression

Transcriptome data were compared between the noninoculation and strain SR-9 inoculation treatment groups of sweet sorghum. We performed quantitative real-time PCR to validate the RNA-seq gene expression analysis results. Three genes, LOC8078735, LOC8055698 and LOC8085850, which may play important roles in the IAA signal response pathway, were confirmed by qPCR and showed a high correlation (R^2^  =  0.94), confirming the reliability of the RNA-seq data.

A total of 1206 differentially expressed genes (DEGs) were obtained from sweet sorghum inoculated with strain SR-9, and Gene Ontology (GO) functional annotation was performed. A total of 1128 genes were annotated to 11 different molecular functions. Among them, the DEGs with catalytic activity were the most abundant. A total of 1271 genes were annotated to 12 different cellular components, of which the top three in abundance were the cell part, cell membrane part, and organelle. A total of 919 genes were annotated to 17 different biological processes, with the most genes annotated to cellular processes. The DEGs of the strain SR-9 inoculation group under Cd stress reflect the molecular mechanism by which strain SR-9 promotes sorghum growth. Therefore, GO enrichment analysis was performed on DEGs in the strain SR-9 treatment group, and the term jasmonic acid hydrolase was the most enriched (Padjust: 0.0113378187378). There were additional DEGs annotated to catalytic activity, adhesion, cell part, and cell membrane part.

**Figure 8:**
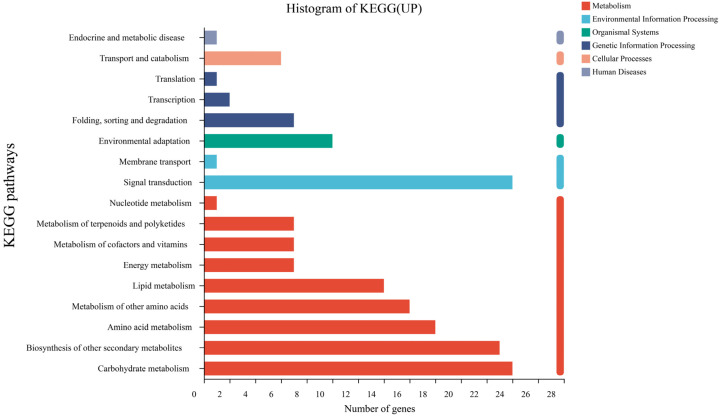
Key function annotation of upregulated KEGG pathway genes between the G20 (noninoculation) and G20S (strain SR-9 inoculation) treatments.

**Figure 9:**
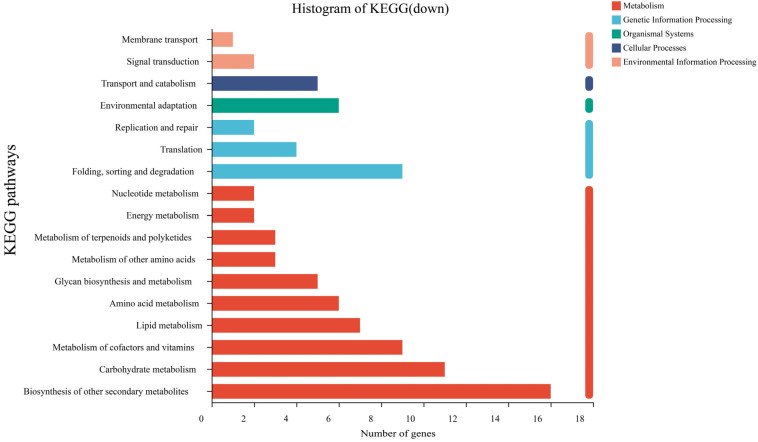
Key function annotation of downregulated KEGG pathway genes between the G20 (noninoculation) and G20S (strain SR-9 inoculation) treatments.

A total of 198 upregulated DEGs and 93 downregulated DEGs in sweet sorghum inoculated with strain SR-9 under Cd stress were annotated to six KEGG metabolic pathways, including genetic information processing, biological system, metabolism, environmental information processing, cellular process, and human disease (Figure 8 and Figure 9). Most DEGs were annotated in the metabolic category. Among them, the most upregulated genes were involved in carbohydrate metabolism (24 strips), biosynthesis of other secondary metabolites (23 strips), signal transduction (24 strips), amino acid metabolism (18 strips), and lipid metabolism (14 strips). DEGs that were downregulated mainly included biosynthesis of other secondary metabolites (16 strips), carbohydrate metabolism (11 strips), cofactor and vitamin metabolism (9 strips), transport and catabolism (5 strips), and environmental adaptation (6 strips).

To further study the metabolic pathways associated with DEGs of sweet sorghum inoculated with strain SR-9 under Cd stress and conduct pathway enrichment analysis, a total of 124 DEGs were annotated into the top 20 metabolic pathways, which were mainly related to plant metabolic pathways. In particular, the phenylpropane biosynthesis pathway (13 upregulated, 12 downregulated) was followed by phytohormone signal transduction in the signal transduction pathway (20 upregulated, 2 downregulated). Upregulated DEGs in plant hormone signal transduction pathways included plant hormones such as auxin, cytokinin, abscisic acid, ethylene, jasmonic acid, and salicylic acid. After inoculation with strain SR-9, the auxin indole acetate protein, gene LOC8078735, gene LOC8055698, and gene LOC8085850 were upregulated, and the expression of the IAA30 and IAA14 genes, which encode repressors of the IAA signal response pathway, was significantly downregulated. This indicated that the main effects of strain SR-9 inoculation in Cd-contaminated soil were related to pathways such as signal transduction, carbohydrate metabolism, amino acid metabolism, and biosynthesis of other secondary metabolites.

## 4. Discussion

Plant growth and biomass are important indices reflecting soil environmental quality. Previous studies have shown that inoculation with exogenous PGPB can promote plant growth and enhance the ability of plants to accumulate and become enriched in heavy metals [27,28]. Due to the important effects of plant-microbe interactions on plant growth and remediation in the rhizosphere, it is important to explore the effects of the tested strains on microbial community structure and function and plant gene expression during plant-microbial joint remediation.

### 4.1. Strain SR-9 Inoculation Improves Sorghum Biomass and Cd Uptake

The biomass and heavy metal content of repair plants during phytoremediation are important factors affecting remediation. The sweet sorghum used in this study has the advantages of a fast growth rate, large biomass, and high photosynthetic efficiency. The uptake of Cd by sorghum plants was higher than that of maize, barley, sunflower, alfalfa, and other crops in Cd-contaminated soil. Analysis of morphophysiological characteristics showed that sweet sorghum could absorb Cd and other heavy metals, and its growth was not negatively affected by mild stress [24]. When the content of Cd in soil was high, it was conducive to the absorption and accumulation of sweet sorghum [29]. In addition, Yuan et al. [30] studied the planting of sweet sorghum in three polymetal-contaminated soils containing different concentrations of Zn, Pb, and Cd and found that roots are the main organ for heavy metal adsorption into all tissues of sweet sorghum, and hybrid sweet sorghum has a high accumulation capacity for Cd. In the remediation of soil heavy metal pollution by microorganisms combined with plants, the ability of PGPB to tolerate and absorb heavy metals and promote plant growth is of great importance [31]. Previous studies have shown that in soils contaminated by heavy metals such as Cu, Cd, and Zn, PGPB resistant to heavy metals can promote the growth of sorghum and reduce the toxic effects of heavy metals [24]. Inoculation of *Pseudomonas* sp. T07 increased the biomass and heavy metal uptake of sweet sorghum, and the aboveground and belowground biomass of sweet sorghum treated with inoculum increased by 22.6% and 33.3%, respectively, compared to the control group [32]. In this study, strain SR-9 was a plant growth-promoting bacterial strain with the ability to secrete IAA, siderophores, and soluble phosphorus. Similar to the results of previous studies, strain SR-9 inoculation increased sweet sorghum biomass, Cd content, Cd transport, and accumulation compared with no inoculation. These results indicated that strain SR-9 inoculation could effectively improve the biomass of sweet sorghum under Cd stress and increase the uptake and enrichment of Cd in sweet sorghum, possibly because strain SR-9 exploited the interaction between the rhizosphere and plants to enhance the uptake of soil nutrients by sweet sorghum, increasing the mobility of Cd and reducing its toxicity. Thus, strain SR-9 can promote the growth and development of sweet sorghum and improve its resistance to Cd and heavy metal remediation efficiency.

### 4.2. Strain SR-9 Inoculation Changes the Soil Microbial Community Composition

The composition of the soil microbial community is one of the important factors influencing the toxic effects of heavy metals and plant tolerance in the heavy metal remediation process [33]. Many studies have used soil microbial communities to evaluate the ecological status of heavy metal-contaminated soils [34,35,36]. Plant growth largely depends on soil microbial diversity, and rhizosphere microbial communities can assist plants in absorbing nutrients and improve plant stress resistance through some growth promotion mechanisms [37]. The effects of PGPB depend on their versatility to the environment, adaptability, and the ability to colonize and compete with indigenous microorganisms [38]. Compared with the noninoculation treatment, inoculation with strain SR-9 decreased the diversity of sorghum rhizosphere bacteria, and changing the soil community composition significantly increased the relative abundance of Actinobacteria and Firmicutes and significantly decreased the relative abundance of Proteobacteria and Acidobacteria. Actinobacteria and Firmicutes can fix metals to protect plants from peroxidation damage under metal stress [39]. Therefore, the increase in the proportions of Actinobacteria and Firmicutes may be attributed to the enhanced Cd-enrichment capacity of sweet sorghum, which reduces the Cd content in the soil (Figure 1). The study by Li et al. [40] showed that inoculation of *Firmicutes* BS TTL1 in soil directly induced an increase in the Firmicutes ratio and increased alfalfa biomass, similar to the results of this study. That is, strain SR-9 successfully colonized the rhizosphere soil of sweet sorghum and gained an advantage in the competition in rhizosphere and inner niches [41]. Firmicutes have outstanding tolerance to extreme and nutrient-deficient environments and produce a large number of enzymes with strong activity [42]. Furthermore, Actinobacteria are important indigenous microorganisms in soil and are involved in nitrogen cycling. Therefore, the increased relative abundance of Actinobacteria and Firmicutes may have been one of the reasons for the increased biomass of sweet sorghum in the strain SR-9 inoculation treatment group.

### 4.3. Strain SR-9 Inoculation Changes Soil Microbial Function According to Metagenomic Analysis

In this study, we used metagenomics to investigate the changes in soil microbial community function after inoculation of strain SR-9 in Cd-contaminated soil. SR-9 inoculation increased the metabolic function of soil microorganisms in the rhizosphere of sweet sorghum, indicating that strain SR-9 inoculation increased the metabolic capacity of soil microorganisms and that more genes were involved in microbial metabolic pathways under this treatment. In addition to the unknown functional classification, metabolism had the highest total abundance in the two groups of samples, and genes involved in amino acid transport metabolism, energy production and conversion, and carbohydrate transport metabolism were the top three functional genes in relative abundance. Amino acids have been shown to alter the key phenotypes associated with plant root growth and microbial colonization, symbiotic interactions, and rhizosphere pathogenesis and are key agents of soil nitrogen cycling [43]. Amino acid transport metabolism plays a central role in plant resistance to heavy metal stress. Therefore, this may be one of the reasons for the increase in sorghum biomass and total nitrogen content in the soil in the inoculation treatment group. Bhattacharyya et al. [44] found that rhizosphere bacteria that promote plant growth promote the induction of carbohydrate accumulation in plants, thereby increasing the yield of various plants. Islam et al. [45] found that PGPB inoculation improved carbohydrate metabolism reduced by Cr toxicity.

In addition, plants have different coping mechanisms to different environments and secrete a large number of compounds that may require transporters, which accumulate in plant roots and exude into the rhizosphere microbiome. For example, ABC transporters are involved in many biological functions, including bacterial resistance, pheromone secretion, mitochondrial biological function, and heavy metal detoxification [46]. In this study, the metabolic pathways that were significantly different in the untreated group included ABC transporter, beta-lactam resistance, and degradation of aromatic compounds, which may be due to the interaction between sweet sorghum and rhizosphere microorganisms to adapt to environmental stress. There were metabolic pathways such as lysine degradation, terpenoid backbone biosynthesis, and unsaturated fatty acid biosynthesis in the inoculated group, and different metabolic pathways caused different physiological responses. Terpenoids play a role in relieving adversity, maintaining cell membrane integrity, and promoting and defending plant growth [47]. The results observed for the inoculation treatment group could be attributed to the growth-promoting properties of strain SR-9 affecting plant rhizosphere microbial communities, thereby increasing the relative abundance of metabolic pathways by altering the relative abundance of several metabolic pathways. These metabolic activities may enhance the potential of microorganisms to cope with Cd stress, regulate the relationship between plants and their environment, and alter the uptake of soil nutrients by plants. Thus, the efficiency of Cd remediation in sweet sorghum was improved.

### 4.4. Strain SR-9 Inoculation Changes Gene Expression in Sorghum Roots According to Transcriptomic Analysis

Transcriptome sequencing has been widely used to explore potential key genes and novel genes in metabolic pathways in understudied species. In this study, transcriptome sequencing analysis of the different treatment groups showed that the upregulated expression of genes in the inoculated treatment was higher than that in the uninoculated treatment. Under Cd stress, GO was enriched for DEGs in sweet sorghum, among which jasmonate hydrolase was enriched to the greatest extent, and more DEGs were annotated for catalytic activity, adhesions, cell parts and cell membrane parts (Figure 9). Similarly, colonization of *Arabidopsis* roots by *P. chlororaphis* increases the expression of genes involved in jasmonate, auxin, and salicylic acid synthesis under drought stress [48]. Among the KEGG metabolic pathways, the top 20 genes with significant changes in sweet sorghum were mainly related to plant metabolic pathways, especially the phenylpropane biosynthesis pathway, followed by plant hormone signal transduction, which affects the signal transduction pathway.

Previous studies have found that the phenylpropane biosynthesis pathway can be activated by various abiotic stresses and plays a crucial role in plant response mechanisms; for example, the phenylpropane biosynthesis pathway is involved in the biosynthesis of guaiacol and lignin, which are key components in the angiosperm cell wall [49]. Phenylpropionate biosynthesis was the only enriched pathway in Cd-treated wheat seedling roots. Genes encoding enzymes such as PAL, 4CL, CCR, CALDH, and CAD are involved in the synthesis of flavonoids, isoflavones, and lignin, which may be related to Cd tolerance [50]. Under lead stress, phenylpropane biosynthesis (Ko00940) and phenylpropane metabolism (Ko00360) in *Miscanthus* root were the most abundant pathways among the DEGs [51]. In this study, after inoculation with strain SR-9, gene expression in the roots of sorghum was changed, the ability of sweet sorghum to respond to Cd stress was improved, and its resistance was improved. It is necessary to further determine the role of the inoculated *Brevibacillus* species and their related genes in the remediation of soil Cd pollution.

## 5. Conclusions

This study showed that inoculation with the plant growth-promoting bacteria *Brevibacillus* sp. SR-9 significantly improved the uptake and accumulation of Cd in sweet sorghum plants and promoted the growth and development of sorghum to some extent. Sorghum root was the major organ that accumulated Cd. Strain SR-9 changed the microbial community composition of sweet sorghum rhizosphere bacteria under Cd stress and increased the relative abundance of bacterial genera in sorghum rhizosphere soil. In addition, the increased relative abundance of metabolic genes in soil samples inoculated with strain SR-9 may improve microbial tolerance to Cd, and the functional genes and metabolic pathways of microorganisms in soil changed with the change in the microbial community, which changed the gene expression of sorghum roots and improved the response ability of sweet sorghum to Cd stress. In this study, the microbiological and botanical mechanisms by which strain SR-9 improves sorghum tolerance and heavy metal enrichment were analyzed by combining amplification sequencing and metagenomic and transcriptomic sequencing, providing a theoretical and experimental basis for the joint remediation effect of plants and plant growth-promoting bacteria.

## Data Availability

The data that support the findings of this study are available on request from the corresponding author.
